# Post-attachment neutralization of HPV16 by antibodies derived from Gardasil-vaccinated women

**DOI:** 10.1038/s41541-025-01286-8

**Published:** 2025-11-20

**Authors:** Patricia M. Day, Cynthia D. Thompson, Erin M. Scherer, Joseph J. Carter, Denise A. Galloway, Douglas R. Lowy, John T. Schiller

**Affiliations:** 1 Laboratory of Cellular Oncology, National Cancer Institute, National Institutes of Health, Bethesda, MD USA; 2 Division of Infectious Disease, Department of Medicine, Emory University School of Medicine, Atlanta, GA USA; 3 Fred Hutchinson Cancer Center, Seattle, WA USA

**Keywords:** Human papilloma virus, Protein vaccines

## Abstract

HPV vaccines exhibit high type-specific and antibody-mediated protection against anogenital infection, even after a single dose. Complete and long-term “sterilizing” immunity against incident infection appears to be established in most HPV vaccinees, suggesting that not only are persistent levels of virus-inhibiting antibodies routinely generated but that they are also exceptionally potent at preventing infection. The process of HPV infection is unusually protracted at several steps, including slow internalization after the virions bind to the cell surface. This observation prompted us to comprehensively evaluate the ability of neutralizing antibodies to prevent infection subsequent to HPV pseudovirion attachment to cells. Using sera and memory B cell-derived monoclonal antibodies from Gardasil-vaccinated women, we observed almost complete post-attachment neutralization of HPV16 pseudovirion infection of HaCaT cells three hours after attachment, even when vaccinees’ sera were diluted 250-fold, with a gradual loss of activity up to 18 h. Unexpectedly, three distinct mechanisms of post-attachment neutralization were discovered, capsid shedding from the cell surface, capsid retention on the cell surface, and rapid capsid degradation after internalization.

## Introduction

Prophylactic HPV vaccines are composed of virus-like particles (VLPs) of the major virion protein, L1. They have demonstrated exceptional potency in preventing incident persistent anogenital infection by the vaccine-targeted types and consequently protection from cervical precancer and cancer by those types^1^. Unexpectedly, they not only protect from neoplastic disease but also routinely produce sterilizing immunity, in that most vaccinees never test positive for the targeted types in sensitive viral DNA amplification assays. Notably, it was demonstrated in clinical trials that efficacy against incident persistent infection improved with years since vaccination. This implies that the relatively few “breakthrough” infections, predominately detected soon after vaccination, are likely to be emergence of prevalent infection and not true incident infection^2^.

Several factors likely contribute to the high efficacy and effectiveness of the vaccines, even when delivered in a single dose. Certainly, one critical factor is the ability to consistently induce high levels of type-restricted neutralizing antibodies that stabilize at a relatively high plateau level for more than a decade. However, virologic factors related to the unique mechanism of HPV infection are likely to be equally important^3^.

In a mouse cervicovaginal challenge model, we previously showed that HPV pseudovirions (PsV) are unable to bind and infect intact squamous or columnar epithelium which therefore acts as a barrier to infection^4^. Instead, they initially attach to specifically modified forms of heparan sulfate proteoglycans (HSPG) on the basement membrane that become accessible due to physical or chemical trauma to the overlying epithelium^5^. This wound-enabled basement membrane binding induces a conformational change in the capsid that exposes the N-terminus of the L2 minor capsid protein, allowing its cleavage by furin, a proprotein convertase, which in turn leads to additional conformational changes that ultimately expose a site on L1 that can bind to a keratinocyte cell surface receptor(s) on basal cells, which support differentiation-dependent virus replication^6^. The first part of this process, which takes several hours, provides a window during which the HPV capsid is potentially susceptible to infection inhibition by vaccine-induced antibodies prior to reaching the cell surface, and, theoretically, to inhibition while on the cell surface.

Using the in vivo challenge model described above, it was previously demonstrated that relatively high levels of passively transferred immune sera prevented capsid binding to HSPGs on the basement membrane. However, at lower levels of transferred sera, the capsids could bind to the basement membrane HSPGs, albeit at reduced levels, and undergo the initial conformational changes required for furin cleavage of L2. However, no association with the adjacent keratinocytes was detected. Rather, the capsids were found associated with phagocytic cells, mostly neutrophils, in the cervicovaginal mucus. These observations led us to hypothesize that the phagocytic cells were likely engaging the capsid/antibody complexes via Fc receptor interactions and removing the capsids during the extended period when they were bound to the basement membrane^6^.

However, the in vivo model did not allow us to investigate what happens to capsids that have completed the required HSPG-induced conformational changes and have subsequently bound to the basal keratinocytes during the wound healing process. Therefore, we developed a modification of a standard vitro neutralization assay, described below, to examine this question. It is important to emphasize that in vitro infection differs from in vivo infection as, in contrast to keratinocytes in vivo, the HSPG modifications to which the capsids specifically bind are expressed on the surface of most immortalized cell lines^7^. Therefore, in vitro, all of the initial steps in the infectious process can occur on the cell surface^6^, although the capsids can also bind to the extracellular matrix (ECM) secreted by cultured cells. In established HPV neutralization assays, the PsV and antibodies are allowed to interact in solution before the complexes are added to the target cells, typically transformed 293TT cells^8^. In the assay reported herein, we examined post-attachment neutralization in which the PsV are first bound to the cell surface, unbound capsids are removed and then, after various incubation times at 37^0^C, the antibodies are added to the PsV/cell complexes.

For this work we utilized HaCaT cells, a human keratinocyte line frequently used as an in vitro surrogate for basal keratinocytes. We evaluated sera from Gardasil-vaccinated young women as well as a panel of HPV16 VLP-specific monoclonal antibodies (mAbs) derived from their memory B cells for their ability to prevent HPV16 PsV infection over the course of up to 24 h post-attachment. Using immunohistochemical and biochemical approaches, we also evaluated the ability of the antibodies to bind the PsV at various stages of the infectious process and the mechanisms of both pre-attachment and post-attachment neutralization.

## Results

### Standard neutralization assays

The panel of thirteen mAbs evaluated here was previously examined by the Fred Hutchinson Cancer Center authors for their neutralization capacities of HPV16 PsV in the well-established 293TT-based neutralization assay^9,10^. This method entails preincubation of the mAb and the PsV together for 1 h prior to addition to cells. In the current study, the NCI authors obtained comparable results (data not shown), in that nine of the thirteen mAbs efficiently prevented HPV16 PsV infection (listed in Supplementary Table 1). However, we were interested in examining the neutralization of these antibodies in a cell line with greater relevance to with HPV biology. Therefore, we used HaCaT cells, a basal cell-like keratinocyte cell line, for establishing neutralization profiles and in all of the following mechanistic experiments.

**Fig. 1:**
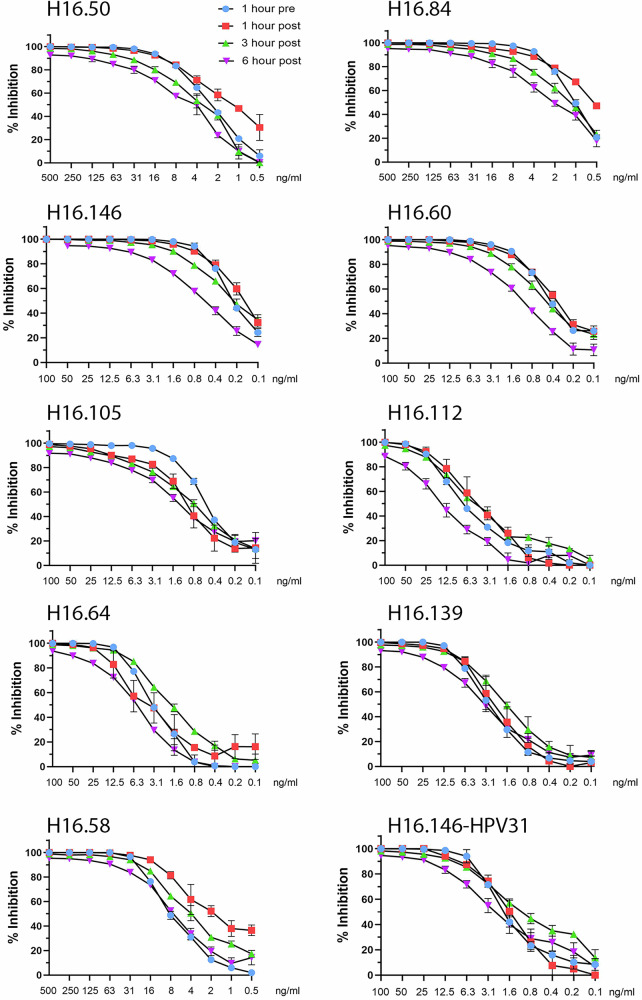
HPV16 neutralization curves for mAb panel. Each mAb that exhibited neutralizing activity against HPV16 in preliminary experiments was examined in a neutralization time course on HaCaT cells. HPV16 PsV expressing GFP was added to HaCaT cells for the indicated times. Following PsV removal and washing, a dilution series of each mAb was added to the final concentration shown on the x-axis. For the pre-attachment condition, PsV and the mAb dilutions were incubated together for 1 h prior to addition to the cells. The final infection time was 72 h. The percentage of GFP-positive cells was determined by flow cytometry and percentage of neutralization calculated by comparison to non-neutralized controls collected for each timepoint (y-axis). Infections were performed in triplicate. The cross-neutralization of HPV31 by H16.146 is shown in the lower right panel. None of the other mAbs showed any capacity for cross-neutralization (See Supplementary Fig. 7A for curves).

We determined neutralization activity when the mAb and PsV were pre-adsorbed, as in the traditional neutralization assay described above. Neutralization activity was also determined at various time points post-PsV attachment to cells. For these experiments, PsV was added to HaCaT cells for the indicated time and the unbound PsV removed prior to addition of the mAb dilution series. Figure 1 shows neutralization curves of the nine neutralizing mAbs when added to HPV16 PsV 1 h prior to addition to cells or following 1 h, 3 h or 6 h of PsV pre-attachment to cells at 37 °C. Interestingly, all of the mAbs retained the ability to efficiently neutralize PsV infection over a prolonged period of time. Notably the highest concentration of all of the mAbs was able to prevent essentially all infection at 3 h post attachment. The four non-neutralizing mAbs described in the 293TT-based assay also had no activity in the HaCaT-based assay (data not shown). Additionally, they showed to protection in vivo using the murine cervicovaginal infection model.

We have tabulated the IC50 calculations for each of the neutralizing mAbs across this time course (Table 1). Remarkably, the IC50s were similar in the 3 h post-attachment assay compared to the pre-attachment assay. Given the strong neutralizing activity even at the 6 h post-attachment time point, we extended the post-attachment time course to 24 h. Surprisingly, we observed at least 50% infection inhibition by all mAbs at the highest concentration at 18 h post-attachment. None of the mAbs generated 50% neutralization in the 24 h assay, although lower level neutralization activity was detected at the highest concentration of all the mAbs. Interestingly, while some mAbs appeared to have a more gradual loss of neutralizing activity across the time series, others appear to lose activity more abruptly at specific intervals, for example, H16.112 between 3 and 6 h, H16.50, H16.105, H16.139, and H16.58 between 6 and 9 h and H16.146 between 12 and 18 h (Supplementary Fig. 1 and Table 1). This observation raised the possibility that the mAbs might have different mechanisms of post-attachment neutralization, a conjecture which we confirmed below.

**Table 1:** mAb IC50s for time course of neutralization

mAb	1 h pre	1 h post	3 h post	6 h post	9 h post	12 h post	18 h post	24 h post
H16.50	2.4	1.3	3.3	5.1	20.7	45.9	18.3	neg
	(2.3–2.6)	(1.1–1.4)	(2.8–3.9)	(4.3–5.9)	(18.4–23.3)	(35.9–61.5)	(12.1–29.5)	
H16.84 (P13B)	1.0	0.5	1.2	1.8	4.2	10.2	5.7	neg
	(0.9–1.0)	(0.5–0.6)	(1.1–1.2)	(1.6–2.1)	(x–4.70	(6.7–14.8)	(5.1–6.5)	
H16.146 (P13B)	0.4	0.3	0.4	1.1	1.9	3.6	34.4	neg
	(0.4–0.5)	(0.3–0.4)	(0.4–0.5)	(1.0–1.1)	(1.6–2.3)	(2.8–4.5)	(24.5–52.9)	
H16.60	0.4	0.4	0.5	1.1	3.5	9.9	4.8	neg
	(0.4–0.5)	(0.3–0.4)	(0.4–0.5)	(1.0–1.1)	(3.1–3.9)	(5.6–21.1)	(3.8–6.1)	
H16.105(P12B)	0.5	1	0.8	1.3	13.9	46.2	16.6	neg
	(0.4–0.5)	(0.9–1.1)	(0.7–0.9)	(1.1–1.5)	(10.4–19.4)	(23.1–x)	(13.3–21.1)	
H16.112 (P13B)	6.4	4.3	4.7	14.2	23.8	71.2	41.7	neg
	(5.4–7.6)	(3.9–4.9)	(3.8–5.7)	(11.7–17.5)	(15.4–39.2)	(46.8–x)	(23.7–79.5)	
H16.64	3.1	4.1	1.7	5.8	16.2	43.8	25.5	neg
	(2.8–3.4)	(3.2–5.2)	(1.6–1.9)	(5.5–6.0)	(12.4–22.0)	(32.8–58.5)	(16.1–42.4)	
H16.139 (P13B)	2.8	2.3	1.6	3.3	24.3	58.6	40.7	neg
	(2.6–3.0)	(2.2–2.5)	(1.6–1.7)	(3.0–3.5)	(19.1–32.1)	(37.8–x)	(24.5–66.0)	
H16.58	7.4	2.1	4.2	7.1	59.5	130	87.1	neg
	(6.5–8.3)	(1.8–2.5)	(3.7–4.7)	(6.4–7.9)	(48.3–75.1)	(120–142)	(68.5–115)	
H16.146-HPV31	3.7	3.1	1.9	4.5	ND	ND	ND	ND
	(3.4–3.9)	(2.9–3.3)	(1.4–2.5)	(3.6–5.6)				

GraphPad Prism software was used to determine IC50 (ng/ml) for each neutralizing mAb at each time point. The 95% confidence interval is noted below each value. “Neg” indicates that an IC50 determination was unstable or otherwise not determinable. Only H16.146 exhibited measurable cross-neutralization against HPV31. “ND” indicates that the time point was not tested.

We also examined the ability of the mAbs to cross-neutralize HPV31 up to 6 h post-attachment. Only H16.146 was found to neutralize HPV31 and was able to do so across the time course examined, with minimal decrease in activity up to 6 h post attachment (final panel of Fig. 1). The HPV31 IC50s were 4 to 10-fold lower than the corresponding HPV16 IC50, but overall, they were well within the range of the HPV16 IC50s for the entire panel (Table 1)

### Immunofluorescent recognition of PsV

HPV capsids undergo a series of conformational changes at various stages of the infectious process (reviewed in ref. ^7^). Therefore, prior to entering into the analysis of the mechanisms by which this panel of mAbs neutralized infection post PsV attachment, we wanted to characterize their ability to recognize HPV16 PsV at various points during this process. PsV particles were added to HaCaT cells for 1 h at 37 °C to allow for initial capsid interaction with the cell surface. All of the neutralizing mAbs were able to strongly recognize cell surface associated PsV at this early time point with the exception of H16.50. Surprisingly the non-neutralizing mAb, H16.133, was also able to weakly bind to capsids adhered to the cell surface. The staining for both of these exceptional antibodies is shown in Supplementary Fig. 2. None of the other non-neutralizing antibodies recognized cell-associated PsV following a 1 h incubation. Additionally, none of the mAbs bound directly to HaCaT cells in the absence of PsV (data not shown). When PsV infection was allowed to continue for an additional 5 h, a strong, specific perinuclear signal was detected for all of the mAbs. Therefore, it seems likely that H16.50 neutralizes PsV following a cell-induced conformational change in the capsid, and the non-neutralizing cohort, H16.133, H16.92, H16.67, H16.110, recognizes either disassembled or partially disassembled capsids in the VLP preparations used to isolate their corresponding memory B cells. Supporting this conjecture, these four antibodies were able to readily recognize HPV16 L1 capsomeric subunits in an ELISA assay (unpublished data). The entire panel of mAbs was also used to stain HaCaT cells following a 24 h infection. All of the neutralizing mAbs, with the exception of H16.105, showed robust, specific staining of PsV, predominantly in a perinuclear pattern reminiscent of the well-described Golgi/ER localization of HPV virions at later time points^11–13^. The detection of PsV at this time point with the H16.105, and the non-neutralizing mAbs H16.92, H16.67 and H16.110, was weak. The distinctive staining observed with H16.50 and H16.133, as noted above, is shown in Supplementary Fig. 2 along with H16.146 as a more typical representative of a neutralizing mAb that recognizes virions throughout binding and endocytosis. As previously described, a subset of the mAbs was also able to detect PsV associated with mitotic chromosomes or within the nucleus^14^.

### ECM binding phenotype

HPV PsV are known to bind the extracellular matrix (ECM) of HaCaT cells via both laminin 332 and HSPGs^15,16^. This binding likely serves as a PsV depot prior to transfer to the cell proper but it is not required for infection. We examined the mAb panel for the ability to detect ECM-associated PsV. PsV was allowed to attach to HaCaT-derived ECM for 3 h at 37 °C, unbound PsV was removed, and mAbs were added for 1 h at 37°C. Following this incubation, ECM was fixed and processed for sequential staining with anti-human IgG (green channel) to detect the bound mAb followed by PsV detection with a rabbit antiserum recognizing HPV16 L1 (red channel). Representative data are shown in panel A of Supplementary Fig. 3. All of the neutralizing mAbs were able to detect bound PsV whereas, none of the non-neutralizing mAbs was able to do so. This experiment also demonstrated the ability of some of the mAbs to competitively prevent binding of the rabbit polyclonal serum. Untreated PsV is shown in the first panel and staining with H16.84, H16.146 and H16.60, as representatives, in the subsequent panels.

In a complementary experiment shown in panel B of Supplementary Fig. 3, we determined if the neutralizing mAb group could prevent PsV association with ECM. PsV and mAb were premixed for 1 h at 4 °C and then applied to the ECM for 3 h at 37 °C. Following this incubation, ECM was fixed and processed for staining with anti-human IgG to detect the bound mAb and anti-laminin 332 to delineate the ECM. **H16.50** and H16.105 both reduced the ability of PsV to bind to the ECM, but the quality of the binding resembled the control whereas, **H16.84** and **H16.146** caused a reduction in ECM binding and the bound PsV displayed a clumped distribution. H16.60, **H16.112**, H16.64, H16.139 and H16.58 all resulted in a complete loss of PsV binding to the ECM. Representative data of mAbs (shown in bold) that permit and prevent ECM binding are shown. The typical distribution of ECM-bound PsV is evident in the upper left image of panel A for comparison. A summary of the results is shown in each panel.

Based on these results, we expect that ECM association would most likely not strongly influence the pre-attachment neutralization assays. However, interpretation of the post-attachment neutralization could be confounded by potential transfer of ECM-bound PsV to the cell surface. Prevention of PsV transfer from this binding depot to the cell surface would be indistinguishable from post-cellular attachment neutralization. To address this issue, we designed an alternative neutralization assay. In this assay, we used EDTA to remove HaCaT cells from their ECM. These suspended cells were incubated with PsV for 3 h in a non-tissue culture treated petri dish. Following this incubation, the suspended cells were pelleted and washed to remove unbound PsV. The cells were then replated to 96 well plates (tissue culture-treated) and the mAb titration added immediately or following attachment for 1 h or 3 h. Thus, the mAbs were added at either 3 h, 4 h or 6 h post-PsV addition. The data for H16.60, H16.112, H16.64 and H16.139 are shown in Supplementary Fig. 4. With the exception of H16.112, IC50s obtained were similar to those shown in Table 1, demonstrating that transfer of PsV from the ECM plays a minimal role in the observed post-attachment neutralization. Therefore, we believe that the sustained ability of the mAbs to prevent infection throughout the time course predominantly reflects neutralization of cell-associated PsV.

### Mechanisms of pre-attachment neutralization

**Fig. 2:**
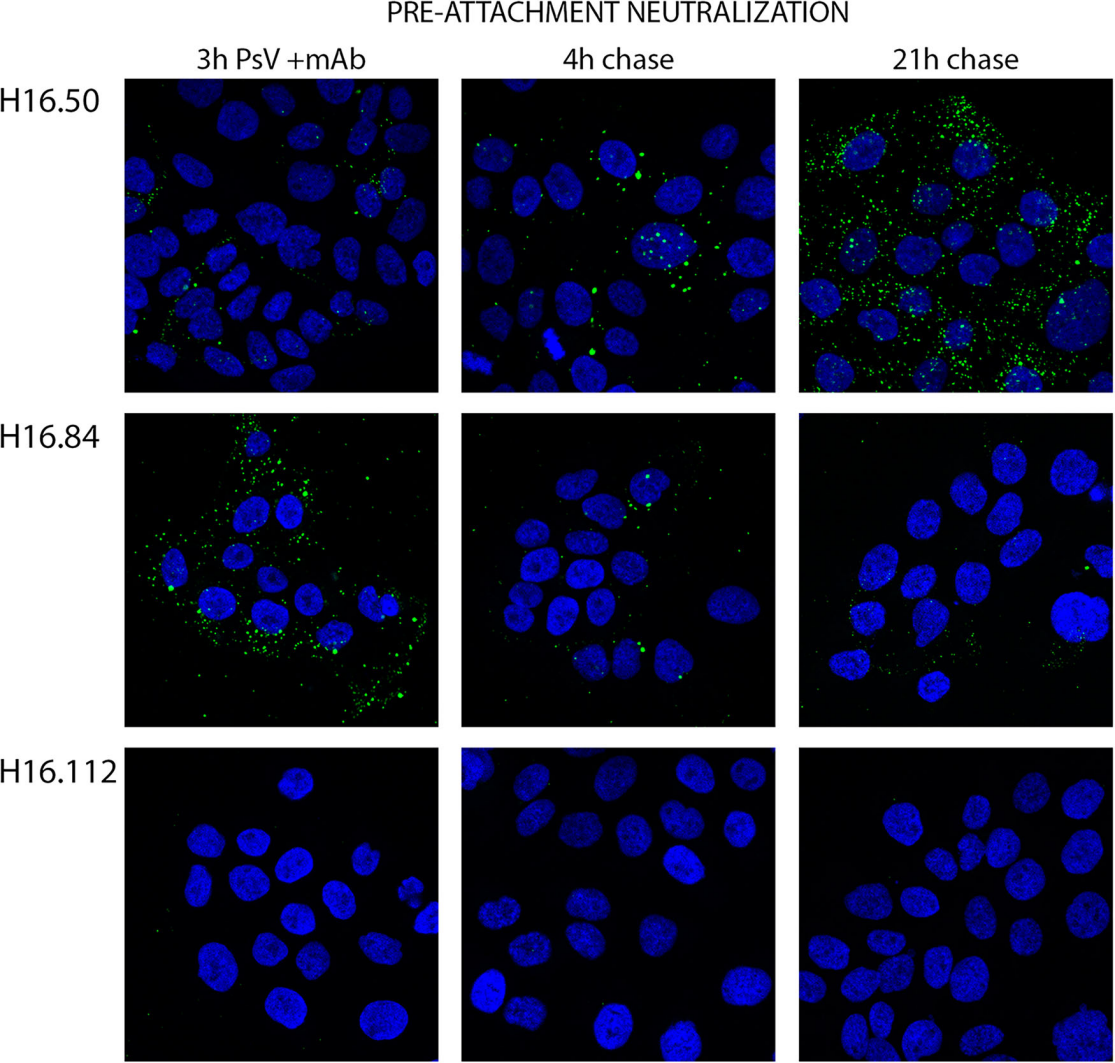
Visualization of pre-attachment neutralization mechanisms. PsV and a neutralizing dilution of each mAb were combined for 1 h and then added to HaCaT cells for 3 h at 37 °C. Unbound PsV was removed by washing and cells were fixed (left column) or incubated for an additional 4 h (center column) or 21 h (right column). Following fixation and permeabilization PsV/mAb complexes were detected with 488-coupled donkey anti-human IgG secondary antibody. Representative staining of the three observed phenotypes is shown. The mAb used is indicated for each row.

We next examined the mechanisms by which the individual mAbs prevented infection when allowed to attach to the PsV prior to and coincident with cell surface association. For this experiment, the mAb and PsV were combined and added together to HaCaT cells for 3 h at 37 °C, unbound complexes were removed, and the cells were either fixed or further incubated for either 4 h or 21 h and then fixed at these chase times. Antibody-bound PsV was detected through use of an anti-human secondary reagent following fixation and permeabilization. Surprisingly, three distinct phenotypic categories could be defined from this evaluation. Selected mAbs (indicated in bold font) representing each phenotype are shown in Fig. 2. The first, defined solely by **H16.50**, showed notable cell surface retention of the PsV throughout the entire 24-h incubation period. Initially the surface distribution was exceptionally clumped but became more diffusely distributed at the latest time point with no ECM association at any point. The second phenotype, observed with **H16.84**, H16.146, H16.60, H16.105, showed initial binding of PsV to cells but then a progressive loss of the signal over the course of the incubation. In the third category, there was no discernible cell-associated or ECM-associated PsV/mAb (**H16.112**, H16.64, H16.139, H16.58) following the initial 3 h incubation nor following either chase. In a parallel experiment, we also detected the cell-associated PsV using a rabbit anti-VLP, in addition to the mAb-bound PsV. The same patterns were observed, ensuring that the diminished signal detected with the anti-human IgG reagent was not simply due to detachment of the mAb from the PsV during the period of the chase (data not shown). Incubation of the mAbs with cells in the absence of PsV did not result in a detectable signal with the anti-human secondary antibody (data not shown).

### Mechanisms of post-attachment neutralization

We next wanted to determine if distinct phenotypes also existed for post-attachment neutralization with these mAbs. For these experiments, PsV was allowed to attach to cells for 3 h at 37 °C, unbound PsV was removed and mAb was added for either an additional 4 or 21 h. Additionally, to distinguish between cell surface antibody detection and intracellular binding, we performed a sequential staining procedure. Following fixation, but prior to membrane permeabilization of the cells, PsV-antibody conjugates were detected with anti-human IgG coupled to Alexa Fluor-488. Thus, only extracellular epitopes would be detected in the green channel. Following removal of unbound 488-coupled antibodies, intracellular epitopes were stained by inclusion of the mild detergent Brij-58 with anti-human IgG- Alexa Fluor-594, allowing the additional detection of intracellular conjugates in the red channel.

**Fig. 3:**
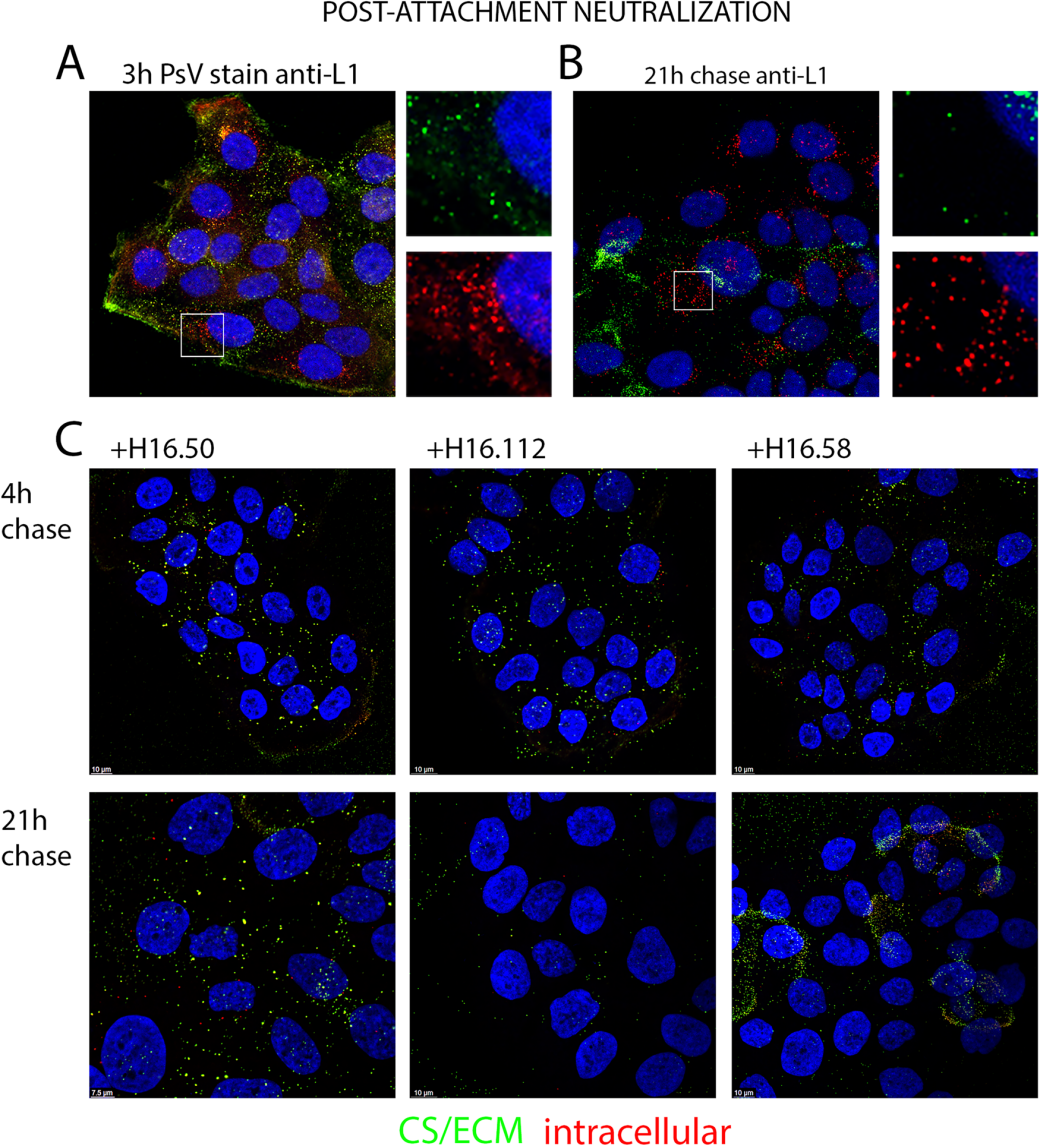
Visualization of post-attachment neutralization mechanisms. HaCaT cells were incubated with PsV for 3 h. Cells were then washed to remove unbound PsV and a neutralizing dilution of each mAb added for the chase time indicated and then fixed. Intracellular and extracellular PsV were differentiated using sequential staining. Extracellular complexes were detected with 488-coupled donkey anti-human IgG following fixation. Subsequently intracellular complexes were detected by incubation with 594-coupled donkey anti-human IgG with the inclusion of 0.1% Brij58 in the dilution buffer. Therefore, cell surface (CS) and ECM-associated PsV fluoresce green and intracellular PsV fluoresce red. Colocalized signal (yellow) indicates extracellular epitopes that were not blocked by incubation with the initial secondary antibody. To control for PsV distribution without antibodies at 3 h and following a 21 h chase, rabbit anti-VLP antiserum staining was performed (panels (**A**) and (**B**), respectively); magnified panels are included. Intracellular signal (red only) was clearly observed at both timepoints. The PsV staining after treatment with a mAbs representing each of the three neutralizing phenotypes is shown below in panel (**C**).

To confirm the utility of this method, infected cells were processed for PsV localization in the absence of the mAbs. The sequential detection with the rabbit anti-L1 serum is shown after the initial 3 h incubation and following a 21-h chase (Fig. 3A, B, respectively). Intracellular virions are detected in the red channel (594) whereas cell surface and ECM bound PsV are detected in the green channel (488) with some coincident red staining due to insufficient occupancy of these epitopes by the application of the first reagent. This experiment illustrates that PsV is distributed throughout the cell by 3 h with staining of both intracellular and cell surface/ECM PsV. As expected, following a 21-h chase period substantially more intracellular PsV was observed, with extracellular capsids predominately bound to the ECM.

We then applied this technique to differentiate extracellular PsV from intracellular PsV following mAb neutralization. PsV was added to the cells for 3 h at 37 °C, then washed and chased for either 4 h or 21 h in the presence of the mAb. We chose a mAb concentration that corresponded to ~90% neutralization (see Fig. 1) to avoid being in substantial antibody excess. We found clearly diminished detection of both cell surface and intracellular PsV following the 4-h chase in the presence of each of the mAbs. However, distinctions among the panel became evident following the longer incubation. Similar levels of PsV were retained on the cell surface for a subset of mAbs (**H16.50**, H16.84, H16.146, H16.60 and H16.105) with a minor intracellular population, whereas for mAbs **H16.112**, H16.64 and H16.139 the PsV was not detectable in either locale at 21 h. An example of these two phenotypes (indicated in bold font) at both chase times are shown in Fig. 3C. Uniquely, the PsV was found predominantly on the ECM after the 21 h chase following incubation in the presence of **H16.58**, which is also shown in Fig. 3.

The observed loss could be indicative of mAb-induced virus shedding from the cell surface and/or increased degradation of intracellular complexes. Some antibody-bound pathogens have been shown to be recognized by the cytosolic antibody receptor, TRIM21^17^. TRIM21 effects antibody neutralization via the degradation of viral capsids and the bound antibody through its action as a ubiquitin E3 ligase that binds the Fc domain of antibodies with high affinity^18^. This action could explain the loss of both mAb and PsV signals, although we do not anticipate that mAb-PsV complexes would traverse the endosomal barrier into the cytoplasm because capsids can be detected within membrane bound vesicles throughout the infectious process^14,19^. Nevertheless, to evaluate the possible involvement of TRIM21-mediated degradation, we examined neutralization in the presence of bortezomib, a proteasome inhibitor. Blocking the action of the proteasome would result in an accumulation of ubiquitin conjugated substrates including the mAb-PsV complex. We compared this treatment with the inclusion of bafilomycin A1 (BafA1), a vacuolar ATPase inhibitor, which would prevent trafficking into the late endosomal/lysosomal compartment and possible subsequent degradation.

**Fig. 4:**
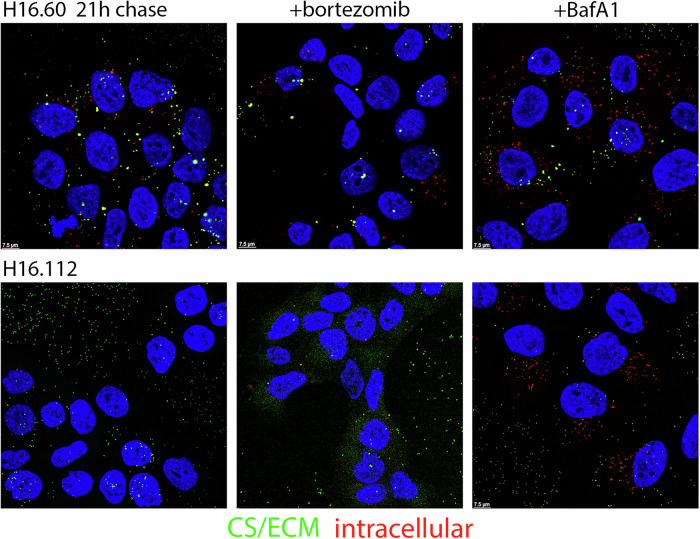
Bortezomib and BafA1 effects on post-attachment neutralization. During post-attachment neutralization, cells were either left untreated or treated with the proteasome inhibitor bortezomib (35 nM) or the vacuolar ATPase inhibitor BafA1 (1 μM). PsV was added to HaCaT cells for 3 h and then chased in the presence of neutralizing mAbs. Detection of PsV-mAb conjugates following a 21 h chase time is shown for neutralization with H16.60 and H16.112. Sequential staining was performed as in Fig. 3. Intracellular staining is detected in the red channel. Cell surface and ECM staining is detected in the green channel.

For the subset of mAbs that inhibited capsid internalization throughout the time course, we found that the intracellular PsV signal (red) was greater in the presence of BafA1 than in the corresponding untreated condition, H16.60 and H16.112 are shown as examples in Fig. 4. Therefore, loss of the mAb-PsV complex is at least partially due to intracellular degradation in an acid-sensitive compartment. We did not observe any rescue of intracellular mAb-PsV complexes following bortezomib treatment (center panel), indicating that TRIM21 was unlikely to be involved in neutralization. A Western blot demonstrating the accumulation of ubiquitinated proteins confirmed the expected action of bortezomib in HaCaT cells (Supplementary Fig. 5).

**Fig. 5:**
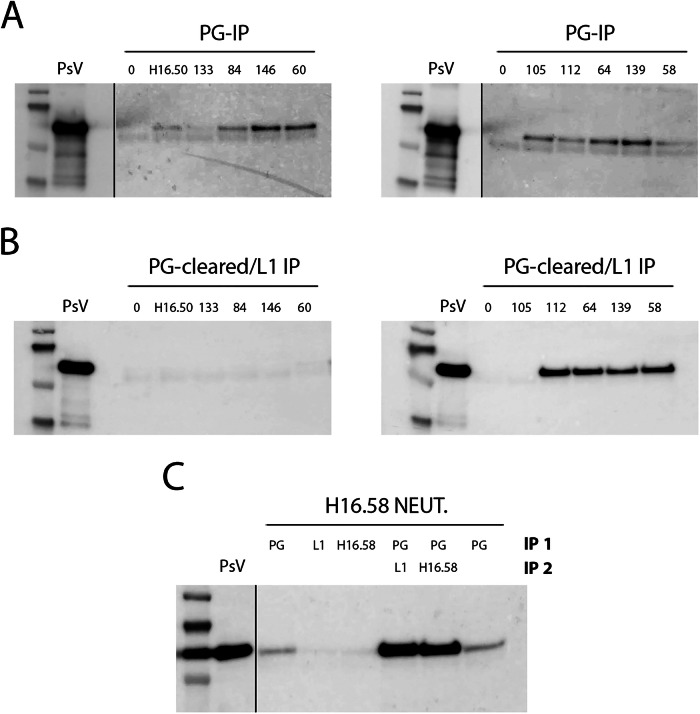
Detection of released PsV-mAb complexes. HaCaT cells were incubated with PsV for three hours and unbound PsV was removed with washing. MAbs were added for an additional 21 h incubation. Culture supernatants were then retrieved and PsV-mAb conjugates were isolated with protein G (PG) magnetic beads, shown in panel (**A**). The PG-cleared supernatants were then immunoprecipitated with the rabbit anti-VLP serum (panel **B**), again using PG beads. PsV was loaded in the first well of each gel to provide an L1 migration control. Panel **C** shows the effect of PG preclearing on the ability to isolate PsV-mAb complexes, using H16.58 as a representative. Cells/PsV complexes were incubated with H16.58 as described and subjected to a first round of immunoprecipitation, indicated as IP1, with either PG alone, rabbit anti-VLP/PG or again with H16.58/PG. The PG immunoprecipitation (duplicated in first and last lanes) replicates what is shown in panel (**A**). The lack of L1 accessibility for immunoprecipitation with either rabbit anti-VLP or H16.58 indicates complete occupancy with the neutralizing H16.58 mAb of available epitopes on the PsV particle. Two PG IP samples were subjected to a second round of immunoprecipitation (IP2) with either the rabbit anti-VLP reagent or H16.58. The ability to isolate L1 from these samples is indicative of the stripping of the H16.58 from the virus by PG treatment, thus rendering these epitopes available for the second IP reaction. L1 was detected with the Camvir-1 antibody.

We next examined the possibility that mAb-induced shedding of cell surface PsV was responsible for the loss of PsV signal seen after engagement with some of the mAbs. For these analyses, PsV was added to HaCaT cells for 3 h at 37 °C. After removal of unbound PsV, mAbs were added for an additional 21 h. Following this incubation, the cell supernatants were recovered and mAb-bound PsV conjugates were isolated with protein G (PG) magnetic beads. No detectable PsV was found in the supernatants of the control cells or cells that were treated with the non-neutralizing mAb, H16.133, indicating little constitutive shedding of PsV from cells. All of these mAbs were shown to be able to immunoprecipitate input PsV (data not shown) and bind to cell-associated PsV at this time point. All of the neutralizing mAbs induced some shedding of the PsV that could be retrieved through PG binding to their Fc domain (Fig. 5A). Additionally, we subjected the PG-cleared supernatant to a second immunoprecipitation with a rabbit anti-VLP serum, shown in panel B. This step was included to capture shed PsV that otherwise would not have been isolated due to potential detachment of the primary mAb following shedding. No PsV was detected in the supernatant without prior mAb treatment confirming that PsV is minimally shed spontaneously from HaCaT cells. Interestingly, including this step revealed that four mAbs, H16.112, H16.64, H16.139 and H16.58, induced high levels of PsV loss from the cell surface, but, probably due to a conformational shift in capsid structure, most of the mAb was dissociated prior to or during the PG immunoprecipitation. In support of this idea, using H16.58 as a representative mAb, we demonstrated that no PsV could be recovered with either the rabbit serum or H16.58 from mAb-treated cells that had not undergone the PG step (panel C). This indicates that H16.58 is complexed with the shed PsV rendering these epitopes unavailable for immunoprecipitation with either the rabbit anti-L1 or with H16.58 itself. Strikingly, after the lysates had undergone the PG immunoprecipitation procedure these epitopes were available for immunoprecipitation with either reagent, indicating that the interaction with PG removed the mAb from the capsids.

The accumulated evidence suggested that multiple mechanisms of post-attachment neutralization are at play and that each mAb seemingly can neutralize by multiple mechanisms depending on whether the PsV is positioned on the cell surface or has already entered the vesicular trafficking pathway. For example, H16.146 and H16.60 may prevent internalization of cell surface PsV and cause degradation of internalized PsV, whereas H16.112 seemingly results in a loss of cell surface PsV in addition to degradation of internalized particles.

We have summarized the mechanisms of both pre-attachment and post-attachment neutralization for each mAb in Table 2. Although the number of mAbs in each category is too low to reach firm conclusions, there did appear to be a tentative correlation between inhibition of capsid binding in the pre-attachment assays and capsid shedding in the post-attachment assays.

**Table 2:** Neutralization phenotypes of mAb panel

mAb NEUTRALIZATION PHENOTYPES	
PRE-ATTACHMENT PHENOTYPES	POST-ATTACHMENT PHENOTYPES
1—cell surface retained	1—cell surface retained
2—progressive loss of cell surface	2—progressive loss of cell surface
3—greatly reduced or no binding	4—degradation

	PRE	POST
**H16.50**	**1**	**1**
**H16.84**	**2**	**1**+4
**H16.146**	**2**	1+**4**
**H16.60**	**2**	1+4
**H16.105**	**2**	1+**2**
**H16.112**	**3**	**2**+4
**H16.64**	**3**	**2**
**H16.139**	**3**	**2**
**H16.58**	**3**	**2**—ECM

A summary of the neutralization phenotypes for both pre-attachment and post-attachment neutralization is shown according to the included key. As described in the text, multiple mechanisms of neutralization were observed for some mAbs. If a dominant mechanism was obvious, it is indicated in bold font. “ECM” indicates that the detached mAb/PsV complex was associated with the extracellular matrix.

### Neutralization profiles of vaccinee sera

**Fig. 6:**
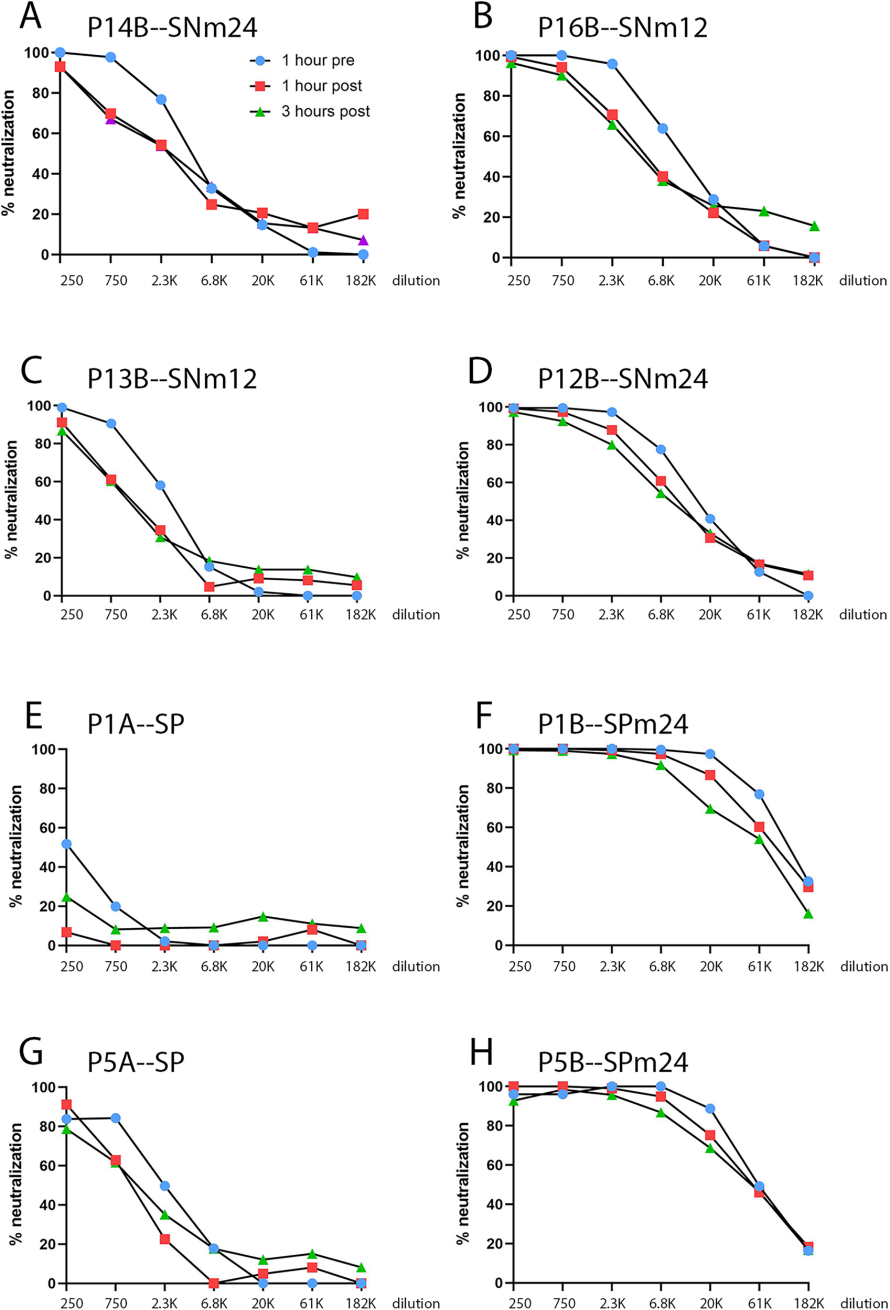
HPV16 neutralization curves for vaccinee sera. The HPV16 PsV neutralization curves across the time course are shown for a subset of vaccinees. Panels **A**–**D** show post-vaccination sera from women that were seronegative upon enrollment. Panels **E** and **F** show matched sera from a woman who was HPV16 seropositive prior to enrollment but essentially lacked neutralizing activity on enrollment (panel **E**) but post-vaccination exhibited high neutralization capacity (panel **F**). Panels **G** and **H** show matched sera from a woman that had been previously exposed and exhibited substantial neutralization prior to vaccination (panel **G**). This neutralization capacity was enhanced following vaccination (panel **H**). In each case the threefold dilution series was started at 1/250. The designations after the serum’s name indicates whether the participant was seronegative (SN) or seropositive (SP) at enrollment and the month following vaccination at which the serum was obtained.

We also performed similar analyses on a panel of sera obtained from vaccinated women^9,10^. We used the participant numbers from the original studies and designated enrollment sera as “A” and sera following vaccination as “B”. This panel includes two participants from which some of the mAbs were isolated. The mAbs H16.84, H16.146, H16.112 and H16.139 all originated from participant 13B and H16.105 was derived from participant 12B^10^. Representative neutralization curves for pre-attachment neutralization and 1 and 3 h post-attachment times are shown in Fig. 6. There were four samples from women that were HPV16 seronegative by ELISA at enrollment^20^. These enrollment sera were also negative for neutralization in our pre-attachment assay (data not shown). The neutralization curves for their post-vaccination sera, P14B, P16B, P13B and P12B, are shown in panels A–D, respectively. The post-vaccination sera, taken either 12 or 24 months after vaccination, neutralized HPV16 PsV infection in both pre- and post-attachment neutralization across the time course. All of these sera exhibited virtually complete neutralization at a 250-fold dilution at all three time points. Therefore, we further tested these four sera across an extended time delay of serum addition, up to 24 h. Remarkably, all of the sera exhibited at least 50% neutralization at 18 h post-attachment, although none of the sera reached this level of activity at 24 h (Supplementary Fig. 6).

Additionally, we obtained sera from eight participants who were HPV16 VLP seropositive upon enrollment. Four of these were then vaccinated and plasma obtained again at 24 months post-vaccination, allowing us to compare the pre- and post-attachment neutralizing activities of the polyclonal antibody response to infection versus vaccination. The other four enrollment-positive participants were not vaccinated, but control plasma was obtained following a 24 month interval. Five of these eight women (P2A, P4A, P5A, P8A and P9A) had enrollment sera that weakly neutralized HPV16 PsV, the other three sera exhibited poor to no neutralization (Table 3). Unsurprisingly, the four enrollment-positive participants that were not vaccinated showed relatively unchanged titers at 24 months, compared to upon enrollment. P9B showed a two- to three-fold increase and the responses of the other three women were generally decreased. In contrast, the four exposed participants that were vaccinated universally showed a dramatic response. The enrollment and post-vaccination curves for a woman with low pre-vaccination titers and a strong response to vaccination, P1A and P1B respectively, are shown in Fig. 6E, F. The responses of a woman with a relatively strong response both pre-vaccination and post-vaccination, P5A vs P5B, are shown in Fig. 6G, H. The IC50s shown in Table 3 demonstrate that the sera from all vaccinated participants show sustained neutralizing activity across the 3 h time course, with IC50s generally decreasing by only 1.1–2.6 fold. Note, a 6 h time point was not performed due to limited sample volumes. Additionally, we evaluated the ability of these sera to cross-neutralize HPV31. Three of the sera, P1B, P5B, and P2B possessed cross-neutralization potential, but only one, P1B, had a high titer. The drop in titers over the 6 h time course was unremarkable compared with those in the HPV16 assay (Supplementary Fig. 7.)

**Table 3:** Vaccinee 50% neutralization titers

Vaccinee	1 h pre	1 h post	3 h post	6 h post	9 h post	12 h post	18 h post	24 h post
P14B (SNm24)	4590	2125	2252	1451	1020	576	113	neg
	(3528–5965)	(1074–4304)	(1287–3709)	(1159–1771)	(920–1124)	(x–1005)	(x)	
P16B (SNm12)	10578	5108	4148	4237	3463	1810	178	neg
	(8663–13006)	(3890–6701)	(3154–5676)	(3473–5210)	(3175–3769)	(1578–2052)	(x)	
P13B (SNm12)	2674	1129	1057	825	607	290	neg	neg
	(2607–2743)	(667–1813)	(892–1237)	(649–1027)	(441–826)	(x–354)		
P12B (SNm24)	15976	9783	8629	6584	5991	2802	225	neg
	(14103–18152)	(9107–10533)	(7624–9814)	(5641–7827)	(4421–8179)	(1910–3836)	(x)	
P1A (SP)	278	214	neg					
	(274–282)	(x)						
P1B (SPm24)	112098	84441	64012					
	(108686–115606)	(81233–87800)	(41972–99435)					
P4A (SP)	1522	934	724					
	(1507–1537)	(782–1099)	(647–805)					
P4B (SPm24)	8542	4518	4517					
	(7840–9334)	(3834–5347)	(3394–5550)					
P5A (SP)	2395	1030	1214					
	(1730–3187)	(715–1469)	(872–1614)					
P5B (SPm24)	59572	52101	50039					
	(50293–72136)	(45982–59569)	(35605–73878)					
P2A (SP)	2638	872	825					
	(2335–2982)	(717–1039)	x					
P2B (SPm24)	90050	82367	76052					
	(88205–92003)	(78899–86000)	(65792–87915)					
P9A (SP)	1354	414	574					
	(1140–1594)	(x–808)	(300–1259)					
P9B (SPm24)	2689	1301	912					
	(2645–2736)	(1069–1558)	(487–1616)					
P8A (SP)	3591	1589	1443					
	(2552–5110)	(1275–1985)	(1038–1939)					
P8B (SPm24)	772	208	263					
	(751–793)	(x)	(x–486)					
P10A (SP)	380	neg	104					
	(368–394)		(x)					
P10B (SPm24)	772	255	219					
	(741–804)	(x–525)	(x)					
P7A (SP)	neg	neg	neg					
P7B (SPm24)	neg	neg	neg					

GraphPad Prism software was used to determine the dilution of each serum that exhibited 50% neutralization at each time point. The 95% confidence interval is noted below each dilution. “Neg” indicates that a titer could not be determined due to low or no neutralizing activity. Only the four sera from previously unexposed women were examined in the extended post-attachment time course. This group is indicated by blue shading. The participants that were ELISA-positive upon enrollment that were subsequently vaccinated are shaded gray. The enrollment positive participants that were not vaccinated are shaded orange. In each instance the participant identifying number is followed by either an A, indicating the enrollment serum, or a B, indicating the 12 or 24 month serum. 12 and 24 are the months since vaccination that the sera was obtained.

*SN* seropositive at enrollment, *SP* seronegative at enrollment.

### Neutralization mechanisms of vaccinee sera

We decided to determine the neutralization mechanisms for these sera. We anticipated that the phenotype of blocking cell surface attachment would predominate, preventing analysis of any additional inhibition mechanisms that the polyclonal sera might possess. That appeared to be the case when we examined the pre-attachment neutralization of the sera from vaccinated subjects, as all of the sera largely prevented PsV binding to the cell surface and ECM (data not shown). However, analysis of the enrollment sera of the naturally exposed patients (that had demonstrable neutralizing titers) revealed that only one of the three had this phenotype (P5A) (data not shown). The other two, P4A and P2A, allowed initial PsV binding but caused a gradual disassociation over the time course (data not shown). Thus, in these instances, vaccination shifted the response phenotype.

**Fig. 7:**
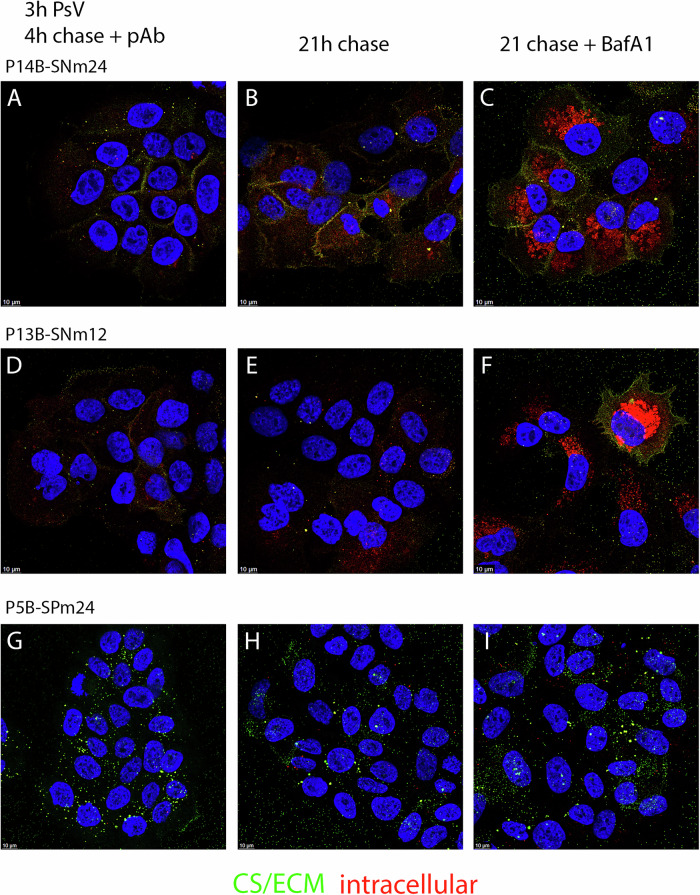
Visualization of post-attachment neutralization mechanisms for vaccinee sera. HaCaT cells were incubated with PsV for 3 h. Cells were then washed to remove unbound PsV, and a neutralizing dilution of each serum was added for the chase time indicated and then fixed. For each serum one condition included a 21 h chase in the presence of BafA1(1 μM). Representative results are shown. Intracellular (red) and extracellular (green) PsV were differentiated using sequential staining as outlined in Fig. 3. Sera from two previously unexposed women are shown in panels (**A**)–(**C**) for P14B and panels (**D**)–(**F**) for P13B. These two women had different phenotypes of post-attachment neutralization as described in the text. Panels **G**–**I** show the phenotype of neutralization for a vaccinated woman (P5B) that had been previously exposed to HPV16 and showed strong neutralization across the post-attachment time course.

Surprisingly, we found more heterogeneity in the post-attachment analysis. Two of the four sera from vaccinated women who were seronegative at entry showed near complete loss of cell surface binding (P16B and P12B) and minimal effect on the quantities of intracellular capsids with BafA1 treatment, indicating that they induced PsV shedding (data not shown). Distinct cell surface staining was retained throughout the incubation with P14B. Interestingly, this pattern was not punctate but clearly defined the cell borders, and incubation in the presence of BafA1 strongly increased the intracellular signal (Fig. 7A–C). This observation likely indicates that the PsV was not shed from the cell surface but rather was efficiently shunted into an intracellular degradation compartment. Cell/PsV complexes incubated with P13B showed little detectable L1 staining, but BafA1 treatment dramatically increased the signal indicating efficient internalization from the cell surface leading to degradation (Fig. 7D–F).

The post-vaccination sera from the women that were seropositive at entry all showed a similar phenotype. The detection of PsV on the cell surface was greatly reduced at the initial timepoint, following a 4 h chase, and this level was retained on the cell surface with no BafA1 effect following the longer chase time. Figure 7G–I shows P5B as a representative of this group. All of the enrollment sera had the same phenotype with the exception of P4A, the enrollment serum for P4B. P4A closely resembled the pattern described above for P14B, with well-defined cell surface staining and an obvious effect with BafA1 treatment. The pre-attachment and post-attachment phenotypes for the vaccinee sera are summarized in Table 4. Most of the sera clearly possessed more than one mechanism of post-attachment neutralization, although one of the mechanisms often seemed dominant. Because inhibition of initial binding was the predominant mechanism of pre-attachment neutralization, no correlation between pre- and post-attachment neutralization mechanisms could be made.

**Table 4:** Neutralization phenotypes of vaccinee serum panel

mAb NEUTRALIZATION PHENOTYPES	
PRE-ATTACHMENT PHENOTYPES	POST-ATTACHMENT PHENOTYPES
1—cell surface retained	1—cell surface retained
2—progressive loss of cell surface	2—progressive loss of cell surface
3—greatly reduced or no binding	4—degradation

	PRE	POST
**P14B**	**3**	1+4*
**P16B**	**3**	**2**
**P13B**	**3**	2+**4**
**P12B**	**3**	1+2*
**P1B**	**3**	1+2*
**P4A**	**2**	**1**+4
**P4B**	**3**	**1**
**P5A**	**3**	1+2*
**P5B**	**3**	1+2*
**P2A**	**2**	1+2*
**P2B**	**3**	1+2*

A summary of the neutralization phenotypes for both pre-attachment and post-attachment neutralization is shown according to the keys. As described in the text, multiple mechanisms of neutralization were observed for most of the sera. If a dominant mechanism was obvious, it is indicated in bold font. The asterisk indicates that a low level of cell surface PsV was maintained throughout the post-attachment analysis even if an additional neutralization mechanism was observed.

## Discussion

In this study, we determined that HPV VLP vaccine-induced antibodies can efficiently prevent infection of HPV16 PsV infection hours after initial cell interaction and that this occurred through multiple mechanisms. These results provide new insights into why the vaccines are so efficacious, inducing sterilizing immunity in almost all vaccinees, even after a single dose. Post-attachment neutralization of HPV PsV was implied in some previous studies based on the ability of antibodies to block infection under conditions that did not block cell association. However, in most of these studies, the antibodies were mixed with the capsids prior to cell exposure or following capsid pre-attachment at 4 °C which would not allow internalization or conformational changes^21,22^. In one exception, the length of time that four murine mAbs could prevent HPV16 PsV infection post-attachment was not determined beyond one hour and the mechanism(s) involved was not evaluated^23^. In a second study, PsV and two neutralizing mouse mAb were shown to internalize together into a nonproductive pathway. However, this phenomenon was also not examined beyond one hour post-attachment^24^.

We decided to examine post-attachment neutralization by both vaccine-induced mAbs and serum antibodies because the antibody repertoire in the memory B cell (mBC) and long-lived plasma cell (LLPC) pools can be distinct, with greater somatic hypermutation, higher avidity, but less diversity in the LLPC pool^25^. Nevertheless, we found that both serum- and mBC-derived antibodies were broadly similar in their relative abilities to accomplish pre-attachment versus post-attachment neutralization over an extended time course. Additionally, we compared post-attachment neutralization profiles of serum antibodies generated after infection versus after vaccination. While the serum IC50s were lower after infection, the relative decay in neutralizing activity observed over the time following PsV binding was similar between the two groups. This was surprising because serum antibodies induced by mucosal infection tend to have lower avidity^26^ and diversity^27^ than those induced by parenteral VLP vaccination.

Only one of the mAbs, H16.146, cross-neutralized HPV31 PsV. Neither the concentration of antibody required for 50% neutralization nor the rate of decay of neutralizing activity after virus binding to the cells was exceptional compared to VLP type-specific neutralization. This was also true for the one vaccinee serum, P1B, that showed strong anti-HPV31 cross-neutralization.

The same three mechanisms of post-attachment neutralization were observed in the antibodies derived from mBCs and sera: namely capsid shedding, cell surface capsid retention, and rapid capsid degradation after internalization. For most of the mAbs and polyclonal sera, more than one mechanism was detected, although one generally predominated (summarized in Tables 2 and 3). Presumptively, multiple mechanisms of post-attachment neutralization by the vaccinee sera are not unexpected. It has been reported that sera generated by most HPV vaccine recipients bind to all five of the major surface loops (although sera generated after infection usually recognized only one)^27^. However, it was surprising that the mAbs were also able to neutralize the PsV by multiple mechanisms since they, by definition, bind a single epitope on the capsid surface. These observations cannot be explained by differences in Fc-mediated interactions as the mAbs are all IgG1 isotypes and differ only in their variable regions^20^.

We previously reported that passive transfer of low amounts of neutralizing anti-VLP serum allowed basement membrane binding in vivo but inhibited detectable binding to keratinocytes^6^. Because the capsids were found to be predominantly associated with neutrophils present in the cervicovaginal mucus, we hypothesized that the HSPG-bound, basement membrane-associated antibody/capsid complexes were phagocytosed by these cells through an Fc receptor-mediated interaction. While this mechanism remains plausible, our current findings suggest two alternative possibilities. The first is that some capsids can reach the keratinocyte cell surface but are then shed when a critical antibody occupancy is reached. The second is that the binding of antibodies to capsids on the cell surface induces sufficiently rapid degradation such that their cell association cannot be detected microscopically. Note that these mechanisms are not mutually exclusive, and we suspect that all three could be at play in vivo, as we have observed in vitro. Although the early processes of capsid rearrangement occur on the ECM and cell surface in vitro versus the basement membrane in vivo, we have found equivalent roles in the infectious process for these locales.

We envision two explanations for the ability of antibodies to interact with and neutralize PsV for such an extended time after cell surface binding. The first is simply that the capsids are retained on the surface for a prolonged period prior to internalization. In this regard, it is well established that HPV capsid internalization is unusually slow and asynchronous (reviewed in ref. ^28^). This is likely due to rate-limiting conformational changes induced by capsid processing by kallikrein 8 and furin^29,30^. Following this asynchronous, stepwise procedure, capsids can be transferred to a secondary receptor and then internalized rapidly^31,32^.

We observed that post-attachment shedding was induced by a subset of mAbs. This could reflect an interruption in the capsid processing at a step which precludes secondary receptor interaction but following a loss of affinity to HSPG, as we hypothesized to occur previously^6,33^. However, it is important to note that our assay cannot distinguish between direct release from the cell surface versus release following rapid endosomal recycling back to the surface. The phenotype characterized by clumped cell surface capsids could be the result of antibody engagement that permits receptor binding but prevents internalization, perhaps by inhibition of interaction with a co-receptor or by inducing aberrant clustering of capsid/HSPG complexes on the cell surface as described for inhibition with the polysaccharide DTSP27^24^.

The second, and not mutually exclusive, mechanism that could explain extended post-attachment neutralization is that pinocytosed antibodies are able to access endocytosed capsids within a vesicular compartment. Antibodies have been shown to reach endosomal compartments by fluid-phase endocytosis and accumulate there if their binding epitope is available^34^. In this study, we clearly observed that a subset of PsV was intracellularly localized by 3 h (Fig. 3). Yet we also found that we could achieve complete neutralization at this timepoint. This observation necessitates that neutralizing mAbs can access this intracellular PsV cohort. Our finding that all of the mAbs were able to bind the capsids after they have undergone endocytosis supports the idea that neutralization can occur after capsid internalization.

The internalization of antibodies following engagement with their cell surface target epitope has been frequently demonstrated (reviewed in ref. ^35^). Therefore, it is possible that antibodies internalized in conjunction with capsids, or accessing their endosomal compartment after capsid internalization, could prevent uncoating events necessary for the retrograde movement from the early endosomes to the Golgi complex. During this transit, the carboxyl terminus of L2 extrudes through the vesicle membrane and engages with cellular proteins that chaperone its delivery to the Golgi (reviewed in ref. ^36^). Prevention of this crucial exposure of L2 could shunt the capsids into a default degradative compartment. Our observation that treatment with the vacuolar ATPase inhibitor BafA1 rescues the internalized capsids is consistent with this possibility.

We did not find evidence that capsid degradation was dependent on TRIM21, the cytosolic Fc receptor and ubiquitin ligase. Specifically, TRIM21-mediated degradation is proteasome-dependent and a proteasome inhibitor, whose activity we validated in HaCaT cells, did not reduce capsid degradation. Although this mechanism of neutralization has been documented for other non-enveloped viruses such as adenoviruses, rotaviruses and picornaviruses^37^, it seemed unlikely to contribute to HPV neutralization because there is strong evidence that HPV capsids remain within membrane-bound compartments throughout the infectious process, including during their association with mitotic chromosomes and even transiently after nuclear entry^14,38^. Our results are in contrast to a report that claimed TRIM21-dependent degradation of HPV capsid/antibody complexes^39^. However, we find it difficult to interpret those findings since the only antibody examined, Camvir-1, is well established to be non-neutralizing and to recognize an epitope that is exposed only in denatured L1.

Post-attachment neutralization has been demonstrated for a number of other viruses, both enveloped and non-enveloped (reviewed in refs. ^40,41^). Mechanisms include prevention of critical conformational changes, binding to endosomal receptors or critical proteolytic cleavages. Exclusively for enveloped viruses, antibodies have been described that inhibit fusion of viral and cell membranes. However, similarly to the HPV neutralization studies discussed above, post-attachment neutralization was only inferred. Either antibody/virus complexes were simply shown to not prevent cell surface binding or the attachment of virus to cells was done at 4 °C to allow adsorption but not further steps in the infectious process. Therefore, the duration of antibody protection post-attachment under physiologically relevant conditions was not evaluated. Examples of the latter include Dengue virus^42^, SARS-CoV-2^43^, Coxsackie A virus^44^, herpes simplex virus^45^, hepatitis C virus^46^, human cytomegalovirus^47^, and Chikungunya virus^48^. In addition, we could find no example in which release of cell-bound virus was induced by the addition of antibody. This might be unique to HPV due to the exceptionally long transit time of the capsids on the cell surface and the complex conformational changes that must occur following binding to the primary HSPG receptor prior to transfer to the internalization receptor.

## Methods

### Pseudovirus production

HPV16 pseudovirus (PsV) were produced according to the improved maturation protocol as previously described and detailed on our laboratory’s website (https://ccrod.cancer.gov/confluence/display/LCOTF/PseudovirusProduction). Briefly, 293TT cells were transfected with a bicistronic plasmid encoding the two HPV16 capsid proteins (p16SheLL), or an equivalent plasmid for HPV31(p31SheLL), together with a reporter plasmid. For evaluation of infectivity by flow cytometry, we encapsidated the reporter plasmid pfwB, that encodes an enhanced GFP protein. For microscopic analysis, the non-fluorescent reporter plasmid, PYSEAP, was packaged. Matured PsV were purified through an Optiprep gradient as described and fractions collected. Protein content of fractions was determined by staining of SDS-PAGE gels with SimplyBlue SafeStain (Novex). Histone containing fractions were combined and quantified according to L1 protein content. PsV infectivity was determined by titration on HaCaT cells. Neutralization assays used a PsV concentration necessary to obtain 20–40% infection at the indicated timepoints.

### Cell lines

HaCaT cells and 293TT were both grown in DMEM media containing glucose and glutamine (Gibco, 11965-092) supplemented with 10% fetal bovine serum and penicillin/streptomycin (Gibco, 15140-122).

### Antibodies

The panel of mAbs were described in two previous publications^9,10^. vaccinee sera were obtained from the same studies. The rabbit anti-L1-only VLP antiserum has been previously described^49^. Camvir-1 (Abcam, Ab-69) was used to detect L1 on Western blots. Mouse anti-GAPDH was purchased from Novus (NBP2-27103). Rabbit anti-laminin 332 was purchased from Abcam (ab14509). Mouse anti-ubiquitin, clone VU1, was purchased from LifeSensors (VU-0101). Alexa coupled secondary antibodies were purchased from Molecular Probes/Thermo Fisher Scientific.

### ECM preparation

For preparation of ECM, HaCaT cells were plated on coverslips as described above, but following overnight incubation, cells were removed by lysis as previously described^50^. For detection of ECM-associated PsV, 50 ng PsV was added to ECM in 500 μl media for 3 h at 37 °C. Unbound PsV was removed by washing and 500 ng of mAb added to the complex for 1 h at 37 °C. Following this incubation the ECM was washed and fixed with 2% paraformaldehyde for 20 min at room temperature. The complexes were detected with donkey anti-human IgG-488. Following this staining, we applied a rabbit anti-VLP antiserum followed by donkey anti-rabbit-594. For evaluation of the ability of each mAb to block PsV association to ECM, we preincubated 500 ng of the mAb with 50 ng of PsV for 1 h on ice in 100 μl. Following this incubation, the volume was increased to 500 μl and added to the prepared ECM for 3 h at 37 °C. Unbound complexes were removed by washing and the ECM was fixed with 2% paraformaldehyde for 20 min at room temperature. The complexes were detected with donkey anti-human IgG-488. Following this incubation, the ECM was delineated by staining with a rabbit anti-laminin 332 antiserum and donkey anti-rabbit-594.

### PsV neutralization assays

HaCaT cells were plated in 96 well tissue culture treated plates at a density of 7 × 10^3^/well and allowed to adhere overnight. For pre-attachment neutralization, the appropriate PsV amount to obtain 20–40% infection in control wells was incubated with a dilution series of each mAb or serum for 1 h on ice. This solution was then applied to the cells and the infection was continued for 72 h. For post-attachment neutralization, the diluted PsV was added to the cells and incubated at 37 °C for the indicated period of time. The equilibration of the PsV amount based on the read-out of infection ensures an approximate equivalence in the ratio of capsids to antibody across the time course analyses. Following this incubation, unbound PsV was removed, and the cells were washed twice in growth media. The dilution series of mAbs or serum was added to the cells following the final wash and infection allowed to continue for 72 h total. For the ECM-free neutralization assay, HaCaT cells were released from tissue culture flasks using 10 mM EDTA/PBS. The cells were added to non-tissue culture treated 6 well plates at a density of 4 ×10^5^/well. HPV16 PsV (1 μg/well) was added to the cells and incubated for 3 h. Following this incubation, cells were pelleted and washed to remove unbound PsV and replated into 96 well tissue culture treated plates, at a density of 7 ×10^3^ cells/well. Cells were either left untreated or mAbs were added in a dilution series as indicated. Following 72 h total infection time the number of fluorescent cells was determined by flow cytometry. Data acquisition was performed on a FACS Canto II flow cytometer (BD Biosciences) following trypsinization and analyzed using Flowjo v10 software (TreeStar). The same preparation of HPV16 and HPV31 PsV were used in all of the experiments to avoid batch variation in the particle to infectivity ratio.

### IC50 determination

The absolute IC50 values for each mAb were determined using the GraphPad Prism software for sigmoidal dose-response with variable slope. 100% infection was considered to be the mean of 8 wells that received PsV only. Cell-only wells served as the negative control. The concentration of each mAb or dilution of each serum that resulted in 50% neutralization was determined. If the response did not reach 50% the IC50 was undefined.

### Detection of PsV and mAbs during the course of infection

HaCaT cells were seeded onto 12 mm high precision glass coverslips (Thor labs 0117520) in 24 well plates at a density of 8 ×10^4^/well, cultured overnight. PsV (40 ng) and antibodies were added to the cells as indicated in the text. For post-attachment analyses, unbound PsV was removed at the indicated times by two washes with growth media. Cells were fixed with 2% paraformaldehyde for 20 min at room temperature, washed three times with 200 mM glycine in PBS and processed for immunostaining. For the determination of PsV detection during a time course of infection, cells were permeabilized with 0.5% Triton X100 in PBS for 5 min at room temperature, blocked for 30 min in 5% normal donkey serum in PBS and incubated with the indicated mAb diluted to 1 μg/ml, followed by staining with 488-conjugated donkey anti-human IgG. For the localization of antibody-PsV complexes following neutralization, cells were either permeabilized with 0.5% Triton X100 in PBS and stained with secondary antibodies or subjected to sequential staining. For the sequential staining procedure, extracellular PsV-mAb conjugates were detected with 488-conjugated donkey anti-human IgG following fixation but without permeabilization. Intracellular complexes were detected with 594-conjugated donkey anti-human IgG diluted with 0.1% Brij58/PBS. In some instances, bortezomib (35 nM) or BafA1 (1 μM) was added during the antibody chase. In all cases, following the final wash, coverslips were incubated with a 250 ng/ml DAPI solution diluted in 0.1% Brij58/PBS for 5 min at room temperature and then inverted onto a drop of Prolong Glass anti-fade mounting solution (Invitrogen P36984) on a glass slide.

### Confocal microscopy

Images were acquired on either a Zeiss 780 confocal system interfaced with a Zeiss Axiovert 100 M microscope or a Leica Stellaris DMi8 system. Images were collated with Adobe Photoshop software. Adjustments were applied consistently across experimental groups.

### Determination of shed PsV

HaCaT cells were plated in 6 well plates at a density of 4 ×10^5^/well. The next day, 500 ng of PsV was added to each well for 3 h at 37 °C. The unbound virus was removed by washing twice. Media was replaced with growth medium containing mAb and incubated for an additional 21 h. Following this incubation supernatants were collected and large cellular debris removed by low speed centrifugation (1200 rpm, 15 min). PsV-mAb complexes were precipitated from the clarified supernatant with 30 μl Protein G Dynabeads (Thermo Fisher 10004D) for 1 h at 4 °C and processed according to the manufacturers’ instructions. In some cases, the supernatant was subjected to a second immunoprecipitation process with either the rabbit anti-VLP serum or H16.58 mAb. Isolated protein was detected by Western blot procedure and L1 was detected with the Camvir-1 antibody.

### Western blotting

Immunoprecipitated protein was released from PG beads by heating in NuPAGE LDS sample buffer (Thermo Fisher NP0008) at 95 °C for 5 min. Proteins were separated on NuPAGE Bis-Tris 4–12% mini protein gels (Thermo Fisher NP0335) and transferred to Immobilon-P membrane (Millipore) and blocked in TBSE-NP40-BSA buffer (50 mM Tris pH 7.5, 150 mM NaCl, 2 mM EDTA, 0.1% NP40 containing 3% bovine serum albumin). For detection of ubiquitinated proteins the membrane was soaked in 0.5% glutaraldehyde in PBS for 20 min prior to antibody incubation. Primary antibodies were diluted in the same buffer. Washes were performed with TBSE-NP40 without BSA. Secondary antibodies, coupled with horseradish peroxidase, were purchased from Thermo Fisher Scientific. Digital gel images were collected on the Amersham Imager 680 system and subsequently processed with the Adobe Photoshop software.

## Supplementary information

supplemental merged

## Data Availability

All data is provided within the manuscript or supplementary information files.
