# Supplementary material for: Post-attachment neutralization of HPV16 by antibodies derived from Gardasil-vaccinated women
Source: NPJ Vaccines. 2025 Nov 20;10:239. doi: 10.1038/s41541-025-01286-8 (PMC12635185; doi:10.1038/s41541-025-01286-8)
Supplement: Supplementary file 1 — supplemental merged [file 41541_2025_1286_MOESM1_ESM.pdf]

SUPPLEMENTAL FIGURE 1

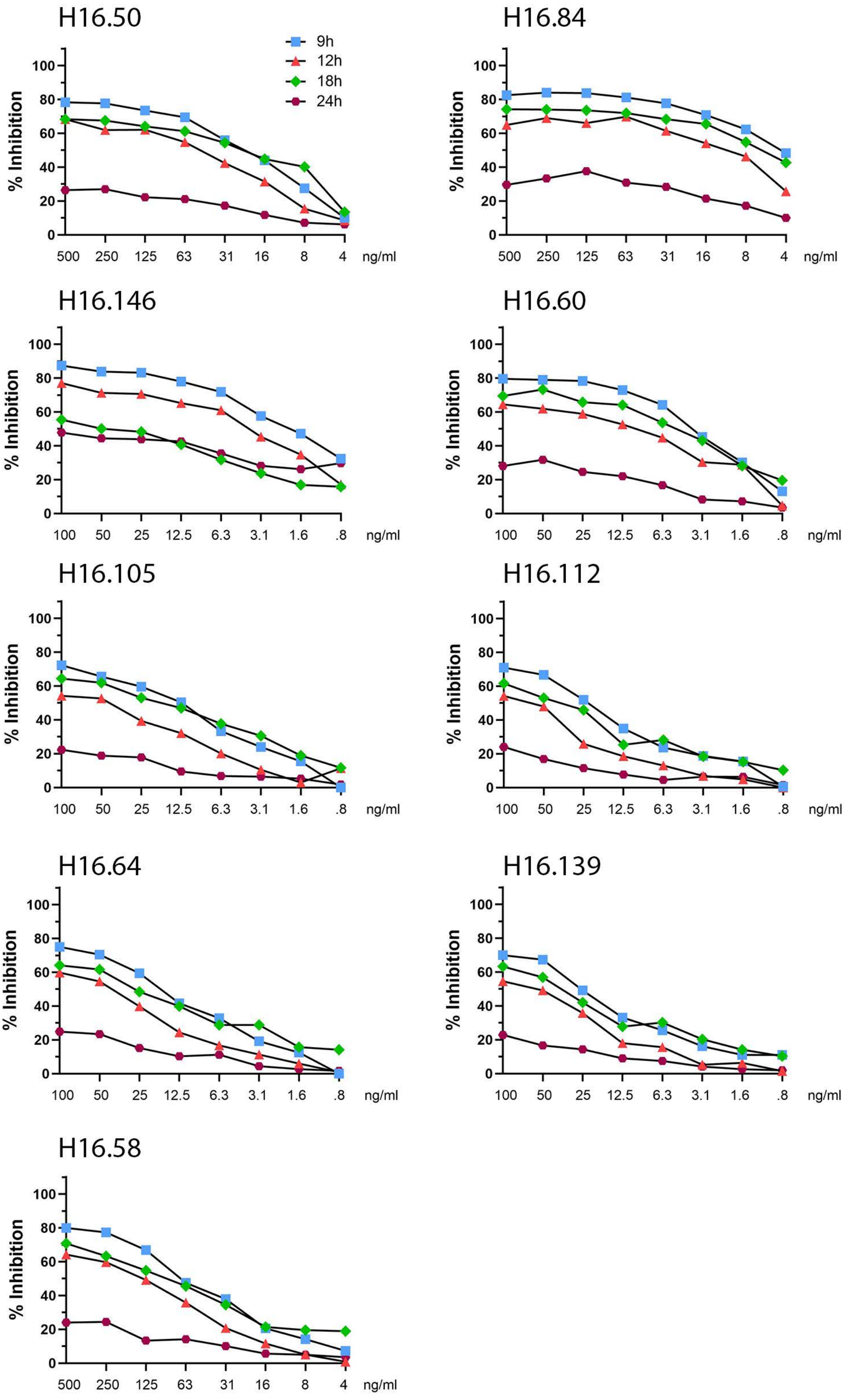

SUPPLEMENTAL FIGURE 2

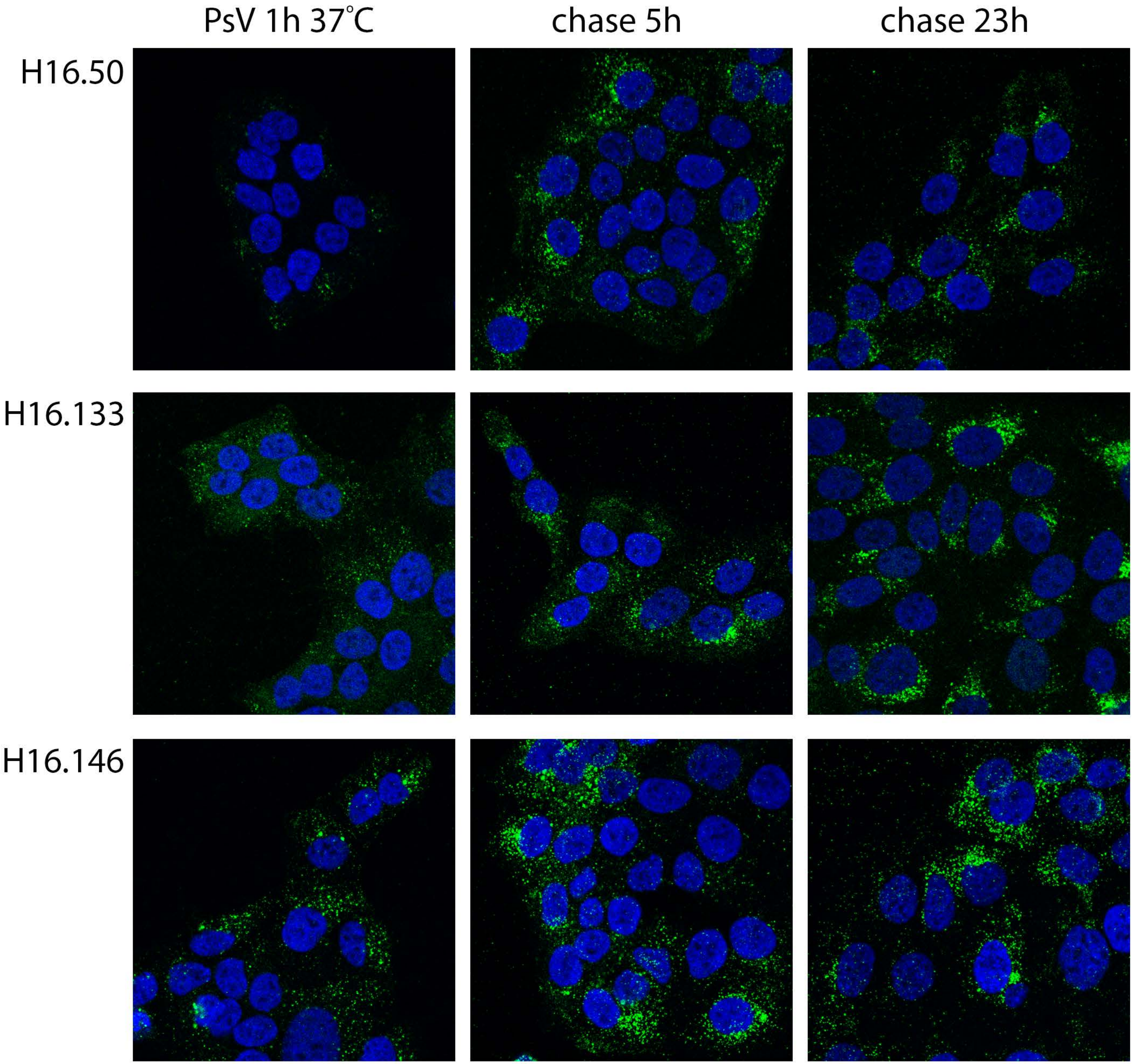

SUPPLEMENTAL FIGURE 3

A ECM staining/rab anti-L1 competition

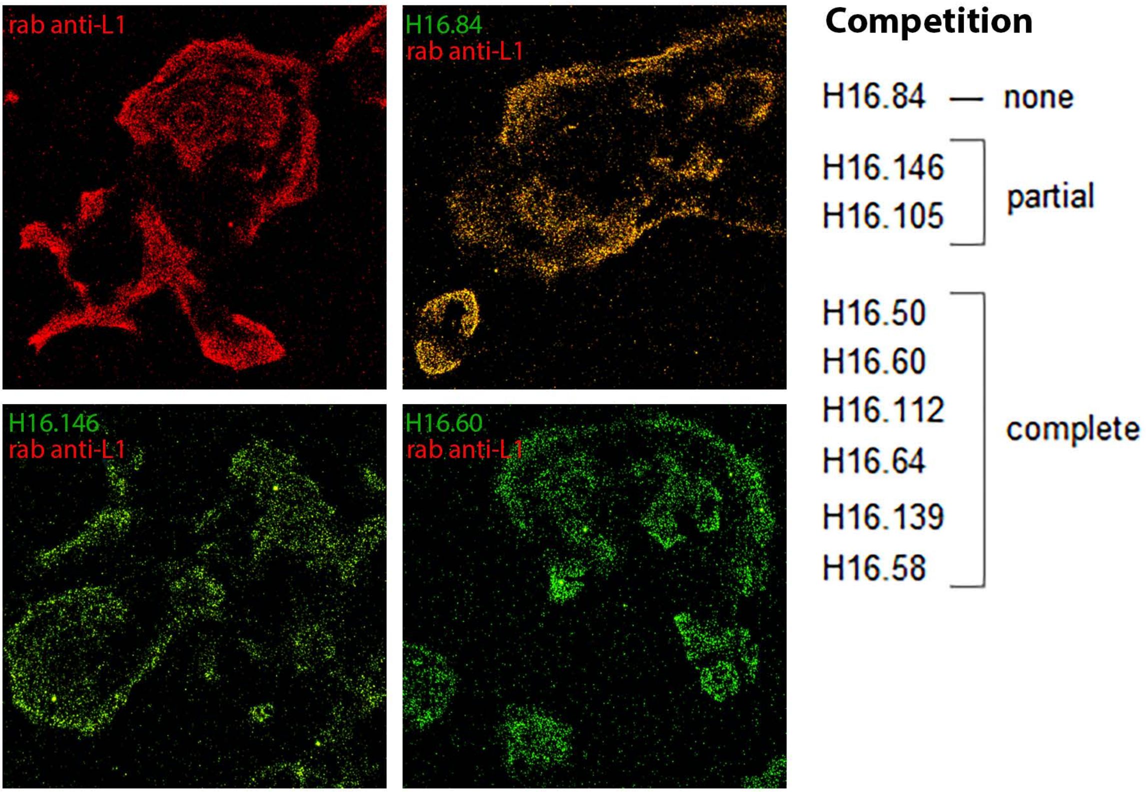

B ECM association

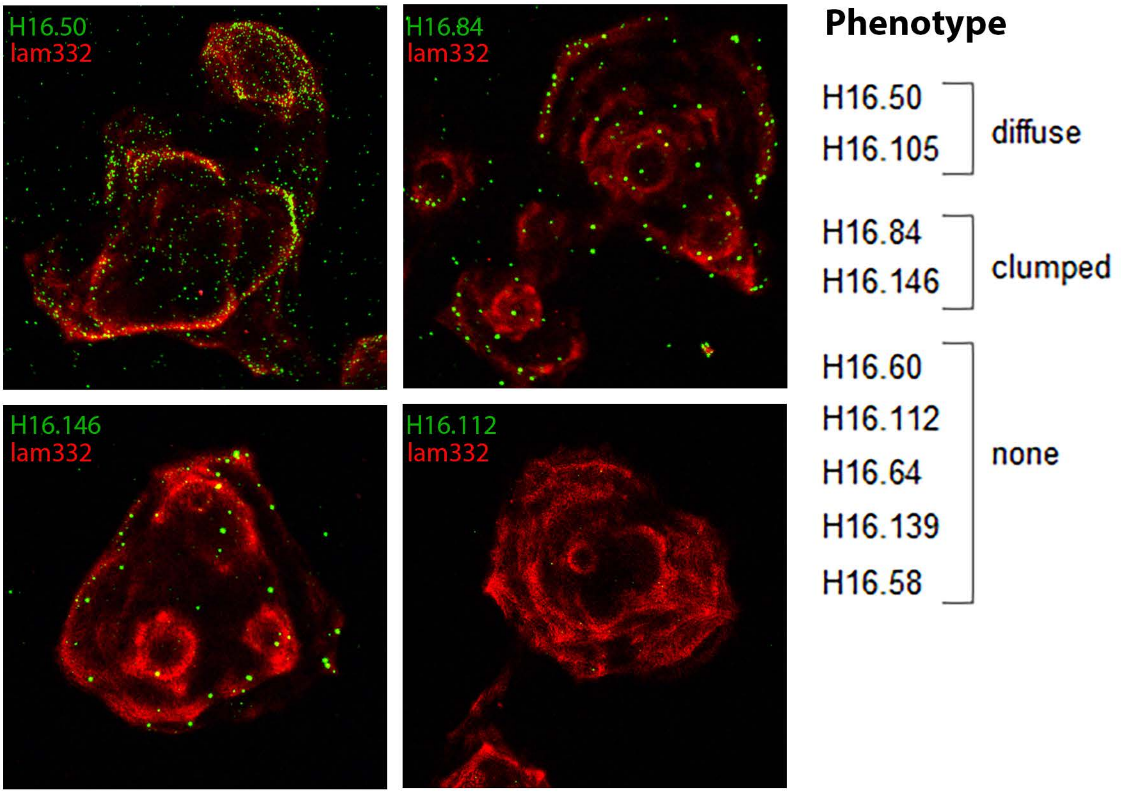

SUPPLEMENTAL FIGURE 4

Alternative neutralization assay

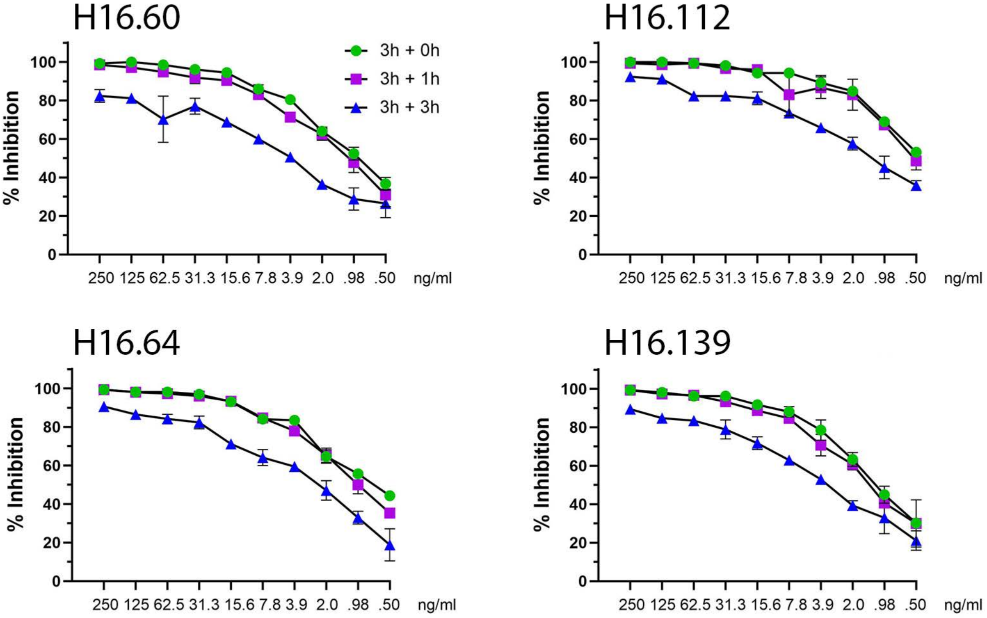

Alternative assay IC50 (ng/ml)

mAb	3h+0h	3h+1h	3h+3h
**H16.60**	**0.9**	**1.1**	**4.2**
	(0.8-1.0)	(1.0-1.2)	(2.8-6.0)
**H16.112**	**0.5**	**0.5**	**1.2**
	(0.4-0.6)	(x-0.6)	(0.9-1.5)
**H16.64**	**0.7**	**1.0**	**2.4**
	(x-0.9)	(0.8-1.3)	(2.0-2.9)
**H16.139**	**1.2**	**1.3**	**3.3**
	(1.1-1.2)	(1.2-1.6)	(2.9-3.8)

SUPPLEMENTAL FIGURE 5

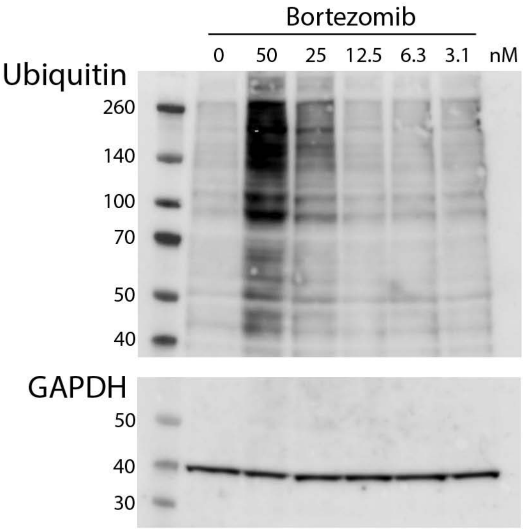

SUPPLEMENTAL FIGURE 6

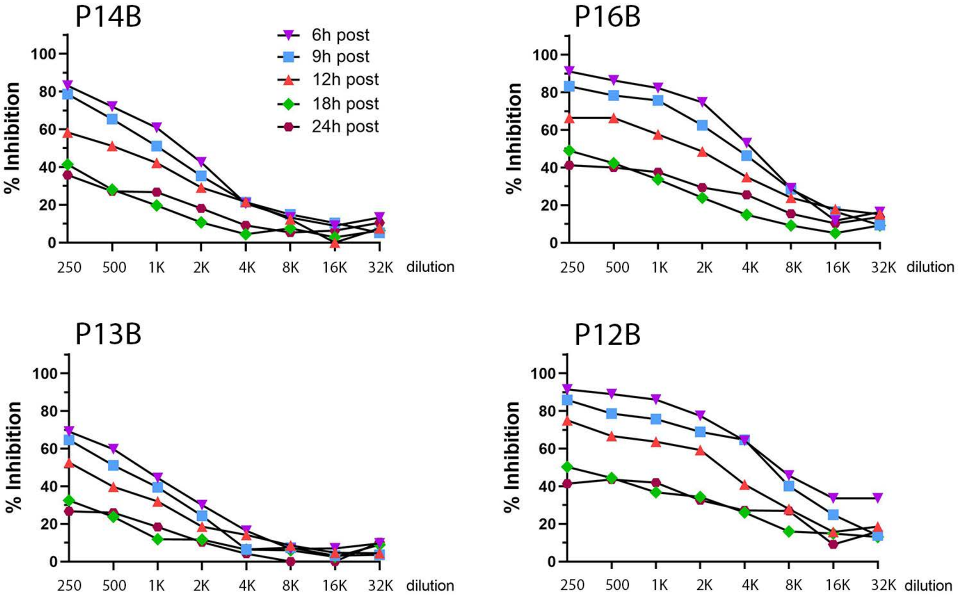

SUPPLEMENTAL FIGURE 7

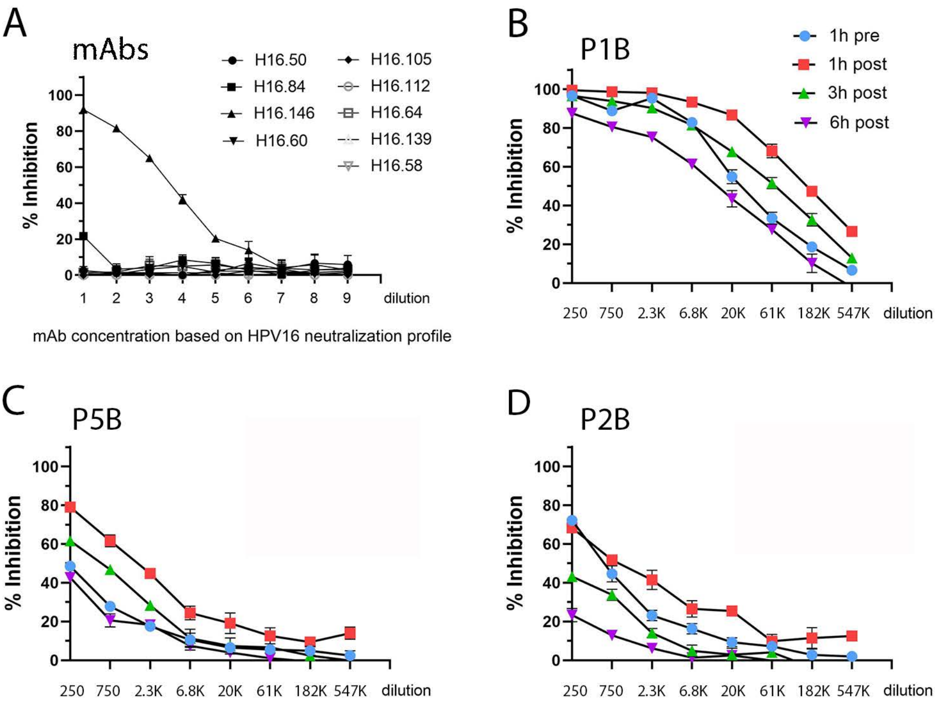

HPV31: 50% neutralization dilution

vaccinee	1h pre	1h post	3h post	6h post
**P1B**	**28672**	**155750**	**66895**	**14365**
	(19417-43987)	(140130-173409)	(61420-73051)	(12224-16780)
**P5B**	**235**	**1512**	**623**	**171**
	(x)	(1101-2004)	(471-829)	(x)
**P2B**	**552**	**975**	**neg**	**neg**
	(460-673)	(516-1856)		

SUPPLEMENTAL TABLE 1

mAb	alt. ID	participant #	
H16.50	7xb14	2	
H16.133	B25M03	12	non-neut.
H16.84	6nv02	13	
H16.146	D25M09	13	
H16.60	7nv10	11	
H16.105	B24.1M01	12	
H16.112	D24.1M02	13	
H16.64	7nv15	11	
H16.139	D25M03	13	
H16.92	B6.1P02	12	non-neut.
H16.67	7nv20	11	non-neut.
H16.110	B24.1P03	12	non-neut.
H16.58	7nv08	11	

Supplemental Figure Legends.

Supplemental Figure 1. Extended HPV16 neutralization curves for mAb panel. HPV16 PsV expressing GFP was added to HaCaT cells for the indicated times. Following PsV removal and washing, a dilution series of each mAb was added to the final concentration shown. The total infection time was 72 hours. At this time, the percentage of GFP+ cells was determined by flow cytometry. Infections were compared to non-neutralized controls collected for each timepoint and the neutralization percentage determined.

Supplemental Figure 2. Detection of PsV entry in HeLa cells. The staining of HaCaT cells with three representative mAbs is shown at three time points post PsV addition. PsV was incubated with cells for 1 hour at 37°C, the unbound removed, and the cells were either fixed and permeabilized at this time (leftmost column) or chased at 37°C for the indicated time, either 6 hours or 24 hours total infection time, and then fixed and permeabilized. PsV was detected with the mAb indicated for each row and 488-coupled donkey anti-human IgG. DAPI staining (blue) was performed for nuclear delineation.

Supplemental Figure 3. Detection of PsV associated with HaCaT cell ECM. Panel A shows the detection of HPV16 PsV bound to ECM derived from HaCaT cells. PsV was bound to cells for 3 hours at 37°C, washed and fixed. PsV was detected with the human mAb listed in each panel followed by staining with 488-coupled donkey anti-human secondary antibody (green). Staining with the rabbit anti-L1 reagent was then performed followed with 594-coupled donkey anti-rabbit secondary antibody (red). The ability of the individual human mAbs to block the subsequent staining with the rabbit anti-L1 reagent is noted on the right. An example of a mAb, as indicated in the panel, exhibiting each phenotype is shown. The upper left panel shows staining with the rabbit anti-L1 serum without any prior mAb staining. Panel B shows the ability of the PsV-mAb complexes to bind to HaCaT ECM. PsV and mAb were incubated together for 1 hour on ice and then transferred to ECM and incubated for 3 hours at 37°C prior to fixation. The human mAb was detected with donkey anti-human-488. The ECM deposition was detected with rabbit anti-laminin 332 antiserum and donkey anti-rabbit-594. The summary of the observed phenotypes is shown on the right and an example of these are shown. The name of the mAb is indicated in each panel.

Supplemental Figure 4. Neutralization of HPV16 in an ECM-free assay. Neutralization of a subset of mAbs was examined following removal of HaCaT cells from their ECM. EDTA-released cells were replated into non-tissue culture treated dishes and HPV16-GFP PsV was added for 3 hours. Cells were collected and unbound PsV was removed by washing. Cells were then replated into 96 well tissue culture treated plates and the mAb dilution series added at the indicated times. GFP positivity was determined at 72 hours post-

infection by flow cytometry. The IC50s for each mAb at each timepoint is shown in the lower panel, with the 95% confidence intervals indicated below each value.

Supplemental Figure 5. Bortezomib effectiveness on HaCaT cells. The optimal concentration of bortezomib to impede proteasomal processing of HaCaT cells was determined. HaCaT cells were incubated for 24 hours with a two-fold dilution series of bortezomib starting at 50 nM. The accumulation of ubiquitinated proteins and the equilibration of total protein were determined by Western blot using antibodies against ubiquitin and GAPDH, respectively.

Supplemental Figure 6. Extended neutralization curves for vaccinee sera. The sera from the four women that were seronegative upon enrollment were evaluated in an extended post-attachment neutralization time course to 24 hours. HPV16-GFP PsV was added to HaCaT cells for the indicated times. Following PsV removal and washing, a dilution series of each serum was added to the final dilution shown. The total time of infection was 72 hours. At this time the percentage of GFP+ cells was determined by flow cytometry. Infections were compared to non-neutralized controls collected for each timepoint and the neutralization percentage determined.

Supplemental Figure 7. Cross-neutralization of HPV31. The cross-neutralization of HPV31 with the panel of mAbs is shown in panel A. The full time course of H16.146 neutralization is shown in Figure 1. The ability of the panel of sera to neutralize HPV31 infection was also determined. The samples that exhibited any positivity, P1B, P5B, P2B are shown in panels B-D, respectively. The 50% inhibitory dilution and the 95% confidence intervals are shown in the lower table. “Neg” indicates that the low level of neutralization prevented this determination.

Supplemental Table 1. mAb panel and naming convention. We have used the mAb designations as previously described in Scherer et al. [9] (H16.50) and Scherer et al. [10] (all others). However, since those publications, the mAbs were assigned an alternative designation (alt ID) as listed in the table based on identifiers used during collection. In this naming convention, the first letter in the previous ID represents the study subject, the following number is the month at which the sample was collected. If the number is a decimal, it was collected one week after the vaccine dose. The letter M or P indicates the source was a memory B cells or plasmablast and the final two digits were added to create unique identifier. The third column indicates the participant number from whose serum the individual mAb was derived [9, 10]. The four non-neutralizing mAbs are also indicated.
